# Elevated circulating GDF11 and its role in age-related sarcopenia: insights from clinical, transcriptomic, and *in vitro* analyses

**DOI:** 10.3389/fragi.2026.1736069

**Published:** 2026-02-25

**Authors:** Rui Chen, Xin Dai, Hong Wang, Ting Zhang, Zhao Zhang, Yaoxia Liu, Zhen Fan

**Affiliations:** 1 Department of Geriatrics, Sichuan Provincial People’s Hospital, University of Electronic Science and Technology of China, Chengdu, China; 2 Department of Critical Care Medicine, Sichuan Provincial People’s Hospital, University of Electronic Science and Technology of China, Chengdu, China

**Keywords:** Gdf11, physical activity, sarcopenia, SMAD signaling, transcriptomics

## Abstract

**Introduction:**

Growth differentiation factor 11 (GDF11), a member of the transforming growth factor-β (TGF-β) superfamily, has been implicated in aging and muscle homeostasis. However, its clinical relevance and mechanistic role in age-related sarcopenia remain incompletely defined.

**Methods:**

Circulating GDF11 levels were quantified in 159 participants stratified by age (<60 vs. ≥60 years) and sarcopenia status. Propensity score matching (PSM) and multivariable logistic regression analyses were applied to identify factors independently associated with sarcopenia. Mendelian randomization (MR) and mediation analyses were conducted to explore potential causal relationships and indirect pathways linking physical activity, circulating GDF11, and sarcopenia. Bioinformatic analyses integrated skeletal muscle transcriptomic datasets and protein–protein interaction (PPI) networks. Mechanistically, differentiated C2C12 myotubes were treated with recombinant GDF11 (rGDF11), followed by assessment of canonical SMAD signaling and muscle atrophy–related markers, including phosphorylated SMAD3 (immunoblotting) and the E3 ubiquitin ligases Atrogin-1 and MuRF1 at both protein (immunoblotting) and transcript (RT–qPCR) levels.

**Results:**

Circulating GDF11 concentrations were significantly higher in older adults than in younger individuals and were further elevated in participants with sarcopenia, both before and after PSM. Multivariable logistic regression identified circulating GDF11 as an independent risk factor for sarcopenia. MR analysis supported a causal protective effect of physical activity on sarcopenia-related traits, while mediation analysis indicated that circulating GDF11 partially mediated this association. Transcriptomic analyses demonstrated that GDF11 mRNA expression in skeletal muscle remained stable regardless of sarcopenia or exercise status, suggesting that elevated circulating GDF11 is unlikely to originate from skeletal muscle. PPI network analysis highlighted enrichment of activin receptor (ACVR)–SMAD signaling pathways. Consistent with these predictions, rGDF11 treatment activated SMAD3 phosphorylation and induced a dose-dependent upregulation of Atrogin-1 and MuRF1 at both the protein and mRNA levels in C2C12 myotubes, supporting activation of a pro-atrophic ubiquitin–proteasome program.

**Conclusion:**

Circulating GDF11 is elevated in individuals with sarcopenia and appears to partially mediate the protective effects of physical activity. Together with functional evidence of activation of catabolic signaling pathways, these findings support a contributory role of circulating GDF11 in age-related muscle loss.

## Introduction

Sarcopenia is a geriatric syndrome characterized by a progressive and generalized reduction in skeletal muscle mass, strength, and function. This condition predisposes older adults to adverse health outcomes, including frailty, falls, hospitalization, disability, and mortality, thereby significantly compromising their quality of life. With the rapid aging of the global population, sarcopenia has emerged as an increasingly urgent public health concern (Sayer et al., 2024). Recent research efforts have focused on identifying systemic biomarkers that can predict or influence the progression of this disorder. Among the potential candidates, growth differentiation factor 11 (GDF11) has attracted considerable interest due to its suggested role in the regulation of aging and tissue regeneration (Fernández-Lázaro et al., 2022; Argentieri et al., 2024).

GDF11, a member of the transforming growth factor-β (TGF-β) superfamily, signals through a receptor system shared with other activin family ligands. Mechanistically, GDF11 initiates signaling through its interaction with activin type II receptors (ActRIIA/B), which subsequently recruit type I receptors (ALK4/5/7) to activate the canonical SMAD2/3 pathway (Egerman et al., 2025; Frohlich et al., 2022). Beyond its involvement in embryogenesis, GDF11 is extensively expressed in adult tissues, functioning as a systemic mediator of inter-organ communication (Frohlich et al., 2022; Lin et al., 2023; Habibi et al., 2024; Bueno et al., 2016). Initially, GDF11 was characterized as a “youth factor,” with preliminary studies indicating a decline in its levels with age and suggesting that supplementation could rejuvenate cardiac and skeletal muscle function (Frohlich et al., 2022; Lehallier et al., 2019; Loffredo et al., 2013; Sinha et al., 2014; Jin et al., 2019). However, this narrative has been contested by conflicting evidence. More recent studies, based on animal and cell-based models, have reported age-associated increases in circulating GDF11 that are associated with reduced muscle mass and strength (Egerman et al., 2015; Wang C. et al., 2024; Hsia et al., 2022). These discrepancies highlight the complex, context-dependent, and potentially deleterious role of GDF11 in muscle aging.

Despite extensive research efforts, the role of circulating GDF11 in age-related sarcopenia in humans remains unclear. To elucidate this ambiguity, we conducted a cross-sectional study to assess the association between circulating GDF11 levels and sarcopenia in an older adult cohort. Concurrently, we analyzed publicly available transcriptomic datasets to examine GDF11 expression profiles in sarcopenic muscle tissue. Additionally, *in vitro* experiments utilizing differentiated C2C12 myotubes were performed to investigate the direct effects of exogenous GDF11 on muscle atrophy.

## Materials and methods

### Study design and participants

This cross-sectional study was conducted at Sichuan Provincial People’s Hospital from January to June 2025. The study protocol was approved by the Institutional Ethics Committee (Protocol No. 2025192), and written informed consent was obtained from all participants. Older adults aged 60–99 years who were capable of completing all physical and clinical assessments were eligible for inclusion. Exclusion criteria were as follows: (1) active inflammatory disease within the preceding 3 months; (2) presence of malignancy, autoimmune disease, liver disease, or renal dysfunction (estimated glomerular filtration rate [eGFR] <60 mL/min/1.73 m^2^); and (3) recent acute cardiovascular or cerebrovascular events. Ultimately, 139 older adults were enrolled and stratified into sarcopenic and non-sarcopenic groups based on established diagnostic criteria (Chen et al., 2020). In parallel, 20 younger adults (aged 18–30 years) matched for sex and body mass index (BMI) were also recruited. Fasting venous blood samples were collected in the early morning and processed for subsequent analyses.

### Sarcopenia assessment and definition

Skeletal muscle mass was assessed using bioelectrical impedance analysis (BIA) (InBody 770, InBody, China). Assessments were performed in accordance with the manufacturer’s instructions: participants stood barefoot on the platform, aligning their heels and toes with the electrodes, and gripped the hand electrodes while keeping their arms slightly abducted to prevent torso contact. The appendicular skeletal muscle mass index (ASMI) was calculated as the sum of the lean mass of the four limbs (kg) divided by height squared (m^2^). Handgrip strength was quantified using a digital dynamometer (EH101, CAMRY, China). Participants performed a maximal isometric contraction using their dominant hand in a standing position with the elbow flexed at 90°. The maximum value from two trials was recorded. Gait speed was evaluated over a 6-m distance. To allow for acceleration and deceleration, participants walked along an 8-m course, and the time taken to traverse the central 6 m (1-m to 7-m marks) was recorded. The faster of two attempts was used for analysis. Sarcopenia was diagnosed according to the 2019 Asian Working Group for Sarcopenia (AWGS) consensus (Chen et al., 2020), defined as the presence of low muscle mass (ASMI <7.0 kg/m^2^ for men, <5.7 kg/m^2^ for women) combined with either low handgrip strength (<26 kg for men, <18 kg for women) or low gait speed (<1.0 m/s).

### Sample size and power analysis

A *post hoc* power analysis was conducted utilizing G*Power software 3.1 to assess the statistical robustness of the primary outcome, circulating GDF11. At a significance level of α = 0.05, the comparison between younger adults (n = 20; 37.96 ± 18.61 pg/mL) and older adults (n = 139; 91.19 ± 46.05 pg/mL) demonstrated a statistical power exceeding 0.99. Within the older adult cohort, the comparison between sarcopenic (n = 98; 108.26 ± 35.55 pg/mL) and non-sarcopenic individuals (n = 41; 84.04 ± 48.23 pg/mL) resulted in a power of 0.86. These findings suggest that the study was adequately powered (power >0.80) to detect clinically significant differences in circulating GDF11 levels.

### Demographic and clinical characteristics

Information regarding socio-demographic characteristics (age, sex, and BMI), lifestyle factors (smoking status and alcohol consumption), and comorbidities (hypertension, diabetes, and hyperlipidemia) was collected. Physical activity was evaluated via the International Physical Activity Questionnaire (IPAQ). Participants were categorized into low, moderate, or vigorous activity groups according to their calculated weekly energy expenditure.

### Measurement of serum GDF11 levels

Serum GDF11 concentrations were measured utilizing a human GDF11 enzyme-linked immunosorbent assay (ELISA) kit (RAB1480, Sigma-Aldrich, United States) in accordance with the manufacturer’s protocol.

### Measurement of vitamin D

Serum 25-hydroxyvitamin D [25(OH)D] levels were quantified using a ELISA kit (SEKH-0341, Solarbio, China) following the manufacturer’s protocol. Vitamin D deficiency (VDD) was defined as a serum 25(OH)D concentration of <20 ng/mL.

### Mendelian randomization analysis

Two-sample Mendelian randomization (MR) analyses were performed to investigate the potential causal effects of physical activity on sarcopenia-related traits using the TwoSampleMR package (Hemani et al., 2017). Summary-level genetic data were retrieved from the IEU OpenGWAS database (https://gwas.mrcieu.ac.uk). The exposure variable was physical activity, defined as strenuous sports participation (ukb-b-151; n = 335,599; 10,894,596 SNPs). Outcome variables included appendicular lean mass (ebi-a-GCST90000025; n = 450,243; 18,071,518 SNPs), usual walking pace (ukb-b-4711; n = 459,915; 9,851,867 SNPs), and low handgrip strength in individuals aged ≥60 years (ebi-a-GCST90007526; n = 256,523; 9,336,415 SNPs). Instrumental variables (IVs) were rigorously selected based on genome-wide significance (*P* < 5 × 10^−9^). To ensure independence, SNPs were pruned for linkage disequilibrium (LD) utilizing a strict threshold of *r* ^2^ < 0.0001 within a 25,000 kb window. To mitigate weak instrument bias, only variants with an F-statistic >10 were retained. Causal estimates were calculated using the Inverse Variance Weighted (IVW) method as the primary analysis. Complementary sensitivity analyses were conducted to ensure robustness, including MR-Egger regression (to detect pleiotropy), weighted median, and weighted mode methods. Heterogeneity was assessed via Cochran’s Q test; if significant heterogeneity was observed (*P* < 0.05), a random-effects IVW model was employed; otherwise, a fixed-effects model was used. Horizontal pleiotropy was monitored using the MR-Egger intercept test. Additionally, the MR-PRESSO framework was applied to detect and correct for potential outliers. Finally, to account for multiple comparisons, the Benjamini–Hochberg false discovery rate (FDR) correction was applied across all exposure–outcome pairs.

### Transcriptomic data analysis

To investigate the transcriptomic landscape associated with GDF11, we systematically searched the Gene Expression Omnibus (GEO) database (https://www.ncbi.nlm.nih.gov/geo/) and identified six datasets meeting the inclusion criteria (Table 1). For sarcopenia profiling, three RNA-sequencing datasets (GSE111016, GSE167186, and GSE226151) were selected using the terms “sarcopenia” and “*Homo sapiens*”, comprising skeletal muscle biopsies from older adults with sarcopenia and age-matched controls. Raw read counts were normalized to transcripts per million (TPM) to correct for sequencing depth and gene length–dependent biases. Differential expression analysis was performed using the limma package (Ritchie et al., 2015). Significant differentially expressed genes (DEGs) were defined as *P* < 0.05 and |log2FC| > 0.5. Functional enrichment of DEGs was assessed using Kyoto Encyclopedia of Genes and Genomes (KEGG) analyses via the clusterProfiler package (Wu et al., 2021), applying an *P* < 0.05.

**TABLE 1:** Transcriptomic datasets.

GEO accession	Sample type	Age (mean ± SEM)	Biopsy tissue	Exercise type	Sample size
GSE111016	Sarcopenia/no-Sarcopenia	72.7 ± 0.9/70.2 ± 0.9	Vastus lateralis	—	20/20
GSE167186	Sarcopenia/no-Sarcopenia	76.8 ± 1.5/72.3 ± 1.3	Lower limb	—	24/29
GSE226151	Sarcopenia/no-Sarcopenia	71.5 ± 1.8/67.6 ± 1.5	Skeletal muscle	—	20/20
GSE226973	Pre/post-exercise	31.5 ± 1.9	Vastus lateralis	Traditional	6
GSE235781	Pre/post-exercise	25.0 ± 1.0	Vastus lateralis	Resistance	8
GSE250122	Pre/post-exercise	22.6 ± 2.8	Vastus lateralis	Acute	8

Given the limited availability of transcriptomic data from sarcopenic individuals undergoing structured exercise interventions, we extended the analysis to healthy young cohorts using the keywords “exercise” and “*Homo sapiens*”. Two RNA-sequencing datasets (GSE226973 and GSE235781) and one microarray dataset (GSE250122) were included. For RNA-sequencing datasets, TPM-normalized expression values were used; for the microarray dataset, normalized expression matrices provided by the original study were analyzed. All exercise-related datasets were derived from vastus lateralis muscle biopsies, a mixed-fiber muscle enriched in type II fibers that is particularly susceptible to age-related atrophy and critical for functional mobility (Nederveen et al., 2020; Prior et al., 2016). Exercise-induced changes in GDF11 expression were assessed using paired t-tests, with *P* < 0.05 considered statistically significant.

### PPI network construction

To construct the protein–protein interaction (PPI) network for GDF11, we integrated data from the STRING (https://string-db.org/) and GeneMANIA (http://www.genemania.org) databases, restricted to *Homo sapiens*. The resulting network was analyzed to identify key interacting proteins. Subsequently, Gene Ontology biological process (GO-BP) enrichment analysis was performed to elucidate the biological pathways and signaling cascades associated with GDF11.

### Protein docking analysis

The three-dimensional structure of the ACVR2B–GDF11 complex was predicted from amino acid sequences using AlphaFold 3 (https://alphafoldserver.com/; UniProt IDs: Q13705 and O95390). The predicted complex was processed using the Protein Preparation Wizard in Schrödinger Suite (2019-01) to correct bond orders and add hydrogen atoms. To resolve steric clashes, energy minimization was performed using the OPLS3e force field with an aqueous solvation model. This optimization followed a two-step protocol comprising steepest descent and conjugate gradient minimization (5,000 iterations each), followed by constrained geometry optimization. Finally, the binding free energy of the complex was calculated using the MM-GBSA method (Sampling: Minimize; Solvation: VSGB; Force Field: OPLS3e). Structural visualization was performed using PyMOL 2.1.

### Experimental cell culture and treatments

C2C12 murine myoblasts (ZQ0092, Zhongqiaoxinzhou Biotech, China) were seeded in culture plates and maintained in growth medium consisting of Dulbecco’s Modified Eagle Medium (DMEM) supplemented with 10% fetal bovine serum, 100 U/mL penicillin, and 100 μg/mL streptomycin at 37 °C in a humidified atmosphere containing 5% CO_2_. When cells reached approximately 70%–80% confluence, the growth medium was replaced with differentiation medium (DMEM supplemented with 2% horse serum and 1% penicillin–streptomycin) to induce myogenic differentiation. The differentiation medium was refreshed every 48 h C2C12 myoblasts were continuously induced to differentiate for 5 days, during which elongated, multinucleated myotubes were formed, consistent with established criteria for myotube maturation (Wang M. Y. et al., 2024; Kim et al., 2025). Physiological circulating GDF11 levels in rodent models are typically reported to range from 2 to 10 ng/mL (Egerman et al., 2015; Kraler et al., 2023; Garbern et al., 2019), and *in vitro* studies commonly employ recombinant GDF11 (rGDF11) at concentrations between 10 and 200 ng/mL (Frohlich et al., 2022; Zhao et al., 2020; Sutherland et al., 2020; Hammers et al., 2017; Sharma and McNeill, 2009; Idkowiak-Baldys et al., 2019; Su et al., 2019). Based on these established conditions, differentiated C2C12 myotubes were treated with rGDF11 (120-11-20UG, PeproTech, United States) at final concentrations of 0–100 ng/mL for 48 h.

### Western blot

Total cellular proteins were extracted from C2C12 cells using RIPA lysis buffer supplemented with protease inhibitors. Cell lysates were incubated on ice for 30 min and centrifuged to collect the supernatants. Protein concentrations were determined using a standard protein quantification assay. Equal amounts of protein were separated by 10% SDS–PAGE and transferred onto polyvinylidene fluoride (PVDF) membranes. Membranes were blocked with 5% skimmed milk for 1 h at room temperature and subsequently incubated with primary antibodies against Smad3 (ab40854, Abcam, United States), phosphorylated Smad3 (p-SMAD3; #9520, CST, United States), Atrogin-1 (67172-1-Ig, Proteintech, China), MuRF1 (55456-1-AP, Proteintech, China) and GAPDH (1E6D9, Proteintech, China), with GAPDH serving as the loading control. After incubation with HRP-conjugated secondary antibodies, protein bands were detected using enhanced chemiluminescence (ECL) reagents and visualized by autoradiography.

### RT-qPCR

Total RNA was isolated using TRIzol reagent (R0016, Beyotime, China) and reverse-transcribed into cDNA using the PrimeScript RT reagent kit (R233-01, Vazyme, China). Quantitative real-time PCR (RT-qPCR) was conducted using SYBR Green Master Mix (Q341-02, Vazyme, China) on a QuantStudio 5 Real-Time PCR System (Applied Biosystems). Transcript levels of Atrogin-1 and MuRF1 were quantified using the 2^−ΔΔCt^ method after normalization to Gapdh, with the control group set to 1. The primer sequences used in this study are listed in Table 2.

**TABLE 2:** Sequence of primers for RT-qPCR.

Genes	Forward primer 5′-3′	Reverse primer3′-5′
GAPDH	CAT​CAA​GAA​GGT​GGT​GAA​GC	AAG​GTG​GAA​GAG​TGG​GAG​TT
Atrogin-1	ACA​TCC​CTG​AGT​GGC​ATC​GC	TGT​AGG​GAC​TCA​CCG​TAG​CG
MuRF-1	TCA​TCC​TGC​CCT​GCC​AAC​A	AGT​AGG​ACG​GGA​CGG​TTG​T

### Statistical analysis

Continuous variables are presented as mean ± standard deviation (SD), unless otherwise specified, and categorical variables as frequencies and percentages. Intergroup comparisons utilized Student’s t-test or the Mann–Whitney U test for continuous data, and the chi-square test for categorical variables. To minimize potential confounding, propensity score matching (PSM) was performed using a 1:1 nearest-neighbor algorithm based on relevant covariates. Variables with a *P* < 0.1 in univariate analysis were advanced to a multivariate logistic regression model. Significant independent predictors (*P* < 0.05) were subsequently used to construct a nomogram for sarcopenia risk prediction. Mediation analysis was executed using Model 4 of Hayes’ PROCESS macro 3.4 for SPSS. To evaluate the significance of indirect effects, bootstrapping was performed with 5,000 resamples to generate 95% bias-corrected confidence intervals (CIs); effects were deemed significant if the 95% CI excluded zero. All analyses were conducted using SPSS 23.0 and R 4.1, with a two-sided *P* < 0.05 defining statistical significance.

## Results

### Elevated circulating GDF11 levels in aging and sarcopenia

**FIGURE 1:**
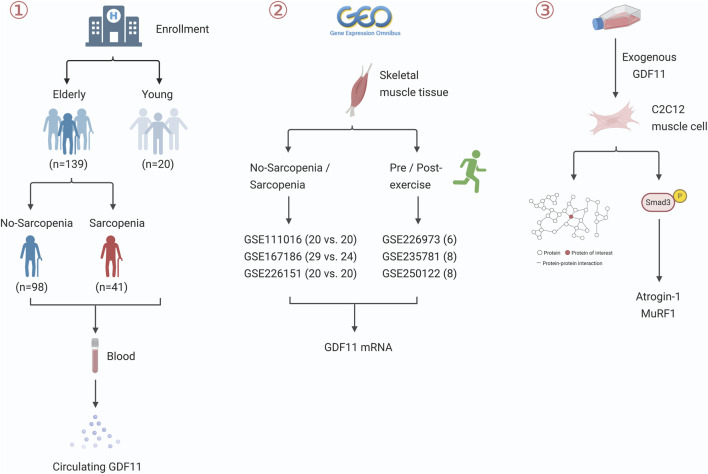
Flowchart depicting the study design.

The workflow diagram for this study is presented in Figure 1. A total of 159 participants were recruited, consisting of 20 younger adults (22.25 ± 3.01 years) and 139 older adults (72.75 ± 6.89 years). Notably, circulating GDF11 levels were significantly elevated in the older cohort compared to the younger group (91.19 ± 46.05 pg/mL vs. 37.96 ± 18.61 pg/mL; *P* < 0.001; Table 3). Subsequently, the older participants were stratified into sarcopenia (SG, n = 41) and non-sarcopenia (NSG, n = 98) groups. The SG group demonstrated expected deficits in clinical characteristics such as gait speed, grip strength, and ASMI (all *P* < 0.001), along with differences in age, sex, BMI, and physical activity (all *P* < 0.05). Importantly, GDF11 levels were significantly higher in the SG compared to the NSG (108.26 ± 35.55 vs. 84.04 ± 48.23 pg/mL; *P* = 0.004). To mitigate potential confounding bias, a 1:1 PSM was performed. Post-matching analysis confirmed that the elevation in GDF11 levels remained significant (107.04 ± 36.83 vs. 74.52 ± 45.53 pg/mL; *P* = 0.003) (Table 4).

**TABLE 3:** General characteristics of all participants.

Variable	Young (n = 20)	Elderly (n = 139)	*P*
Age, years	22.25 ± 3.01	72.75 ± 6.89	<0.001
Male, n (%)	11 (55.0%)	70 (50.4%)	0.698
BMI, kg/m^2^	22.02 ± 2.10	22.72 ± 2.48	0.230
Smoking, n (%)	—	38 (27.3%)	​
Alcohol, n (%)	—	40 (28.8%)	​
Hypertension, n (%)	—	46 (33.1%)	​
Diabetes, n (%)	—	31 (22.3%)	​
Hyperlipidemia, n (%)	—	56 (40.3%)	​
VDD, n (%)	—	35 (25.2%)	​
IPAQ (%)			
Low	—	36 (25.9%)	​
Moderate	—	84 (60.4%)	​
High	—	19 (13.7%)	​
GDF11, pg/mL	37.96 ± 18.61	91.19 ± 46.05	<0.001

**TABLE 4:** Characteristics and GDF11 Levels in elderly with vs. without sarcopenia (pre- and post-PSM).

Variable	Pre–PSM (NSG; n = 98)	Pre–PSM (SG; n = 41)	*P*	Post–PSM (NSG; n = 32)	Post–PSM (SG; n = 32)	*P*
Age, years	71.47 ± 5.10	75.77 ± 8.45	0.004	73.22 ± 5.42	73.59 ± 7.63	0.823
Male, n (%)	44 (44.8%)	26 (63.4%)	0.046	19 (59.3%)	18 (56.2%)	0.800
BMI, kg/m²	22.45 ± 2.25	23.36 ± 2.89	0.048	22.59 ± 2.59	22.81 ± 2.90	0.929
Smoking, n (%)	24 (24.4%)	14 (34.1%)	0.244	9 (28.1%)	10 (31.2%)	0.784
Alcohol, n (%)	26 (26.5%)	14 (34.1%)	0.366	15 (46.9%)	11 (34.3%)	0.309
Hypertension, n (%)	33 (36.6%)	13 (31.7%)	0.822	10 (31.2%)	11 (34.3%)	0.790
Diabetes, n (%)	18 (18.3%)	13 (31.7%)	0.085	11 (34.3%)	10 (31.2%)	0.773
Hyperlipidemia, n (%)	40 (40.8%)	16 (39.0%)	0.844	11 (34.3%)	13 (40.6%)	0.606
VDD, n (%)	22 (22.4%)	13 (31.7%)	0.251	14 (43.8%)	9 (28.1%)	0.193
IPAQ (%)			0.007			0.445
Low	18 (18.4%)	18 (43.9%)		8 (25.0%)	12 (37.5%)	
Moderate	65 (66.3%)	19 (46.3%)		21 (65.6%)	16 (50.0%)	
High	15 (15.3%)	4 (9.8%)		3 (9.4%)	4 (12.5%)	
GDF11, pg/mL	84.04 ± 48.23	108.26 ± 35.55	0.004	74.52 ± 45.53	107.04 ± 36.83	0.003
AWGS criteria						
GS, m/s	1.14 ± 0.44	0.91 ± 0.21	<0.001			
HS, kg	25.95 ± 6.77	19.63 ± 5.14	<0.001			
ASMI, kg/m2	6.85 ± 1.26	5.40 ± 1.29	<0.001			

### GDF11 as a circulating marker of sarcopenia

To identify the clinical determinants of sarcopenia, both univariate and multivariate logistic regression analyses were conducted, as detailed in Table 5. The univariate analysis revealed that advanced age, male sex, and elevated circulating GDF11 levels were significantly associated with increased odds of sarcopenia (all *P* < 0.05), while physical activity was linked to decreased odds (*P* < 0.05). Variables with a *P*-value less than 0.1 in the univariate analysis were included in the multivariate model. Upon adjustment, age (OR = 1.11, *P* = 0.002), moderate physical activity (OR = 0.28, *P* = 0.008), and circulating GDF11 levels (OR = 1.01, *P* = 0.013) remained independently associated with sarcopenia.

**TABLE 5:** Univariable and multivariable logistic regression analysis of clinical factors associated with sarcopenia.

Variable	Univariable OR (95% CI)	*P*	Multivariable OR (95% CI)	*P*
Age	1.10 (1.03∼1.16)	0.001	1.11 (1.04∼1.18)	0.002
Male	2.13 (1.00∼4.50)	0.048	1.50 (0.62∼3.64)	0.368
BMI	1.17 (1.00∼1.37)	0.051	1.05 (0.89∼1.23)	0.580
Smoking	1.60 (0.72∼3.53)	0.246		
Alcohol	1.44 (0.65∼3.15)	0.367		
Hypertension	0.92 (0.42∼1.95)	0.822		
Diabetes	2.06 (0.90∼4.74)	0.088	2.68 (0.95∼7.54)	0.063
Hyperlipidemia	0.93 (0.44∼1.96)	0.844		
VDD	1.61 (0.71∼65)	0.251		
IPAQ		0.009		0.026
IPAQ (Moderate)	0.29 (0.13∼0.67)	0.002	0.28 (0.11∼0.71)	0.008
IPAQ (High)	0.27 (0.07∼0.96)	0.042	0.32 (0.08∼1.37)	0.124
GDF11	1.01 (1.00∼1.02)	0.007	1.01 (1.00∼1.02)	0.013

**FIGURE 2:**
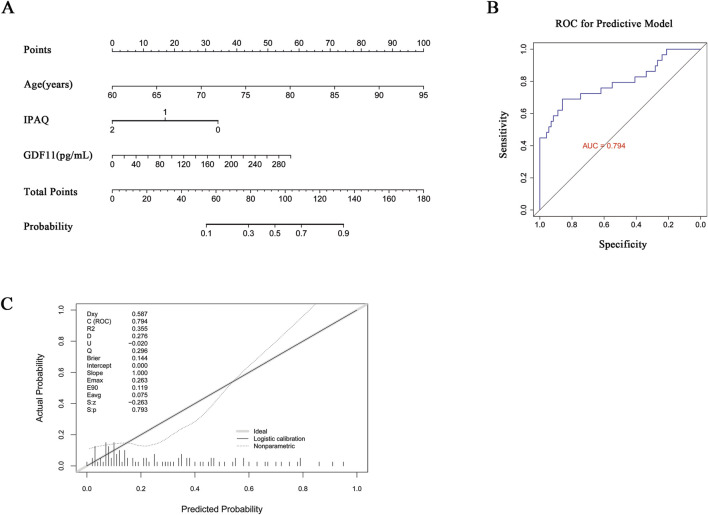
Nomogram-based risk prediction model for sarcopenia. **(A)** A predictive nomogram incorporating age, physical activity, and circulating GDF11 was constructed to estimate the risk of sarcopenia. **(B)** ROC curve demonstrating the discriminatory performance of the nomogram. **(C)** Calibration curve assesses the model’s calibration performance.

A prognostic nomogram was developed based on these independent predictors (Figure 2A). The model exhibited satisfactory discriminative ability, with an area under the receiver operating characteristic curve (AUC) of 0.794 (Figure 2B). Calibration analysis demonstrated a strong concordance between predicted and observed risks (Figure 2C), with a calibration slope of 1.000 and an intercept of 0.000. The model’s fit was further corroborated by a low Brier score (0.144) and a non-significant Spiegelhalter’s p-value (*P* = 0.793). These findings indicate satisfactory internal performance; however, external validation in independent cohorts is warranted to assess generalizability and clinical applicability.

### Physical activity is negatively associated with circulating GDF11 levels

In order to identify factors associated with circulating GDF11 levels, a multivariable linear regression analysis was performed, adjusting for variables including age, sex, BMI, smoking status, alcohol consumption, hypertension, diabetes, hyperlipidemia, physical activity, and VDD (Table 6). Among the covariates analyzed, physical activity emerged as the only factor significantly associated with circulating GDF11 levels (*P* = 0.037). The analysis revealed negative values for both the standardized (β = −0.18) and unstandardized (B = −13.16) coefficients, suggesting an inverse relationship between physical activity and circulating GDF11 levels.

**TABLE 6:** Multivariable linear regression analysis of factors associated with GDF11 levels.

Variable	β	t	*P*	B	95% CI
Constant	​	9.37	<0.001	115.89	91.43∼140.35
Physical activity	−0.18	−2.10	0.037	−13.16	−25.53∼ −0.78

### Circulating GDF11 appears to be partially involved in the protective effects of physical activity against sarcopenia

**FIGURE 3:**
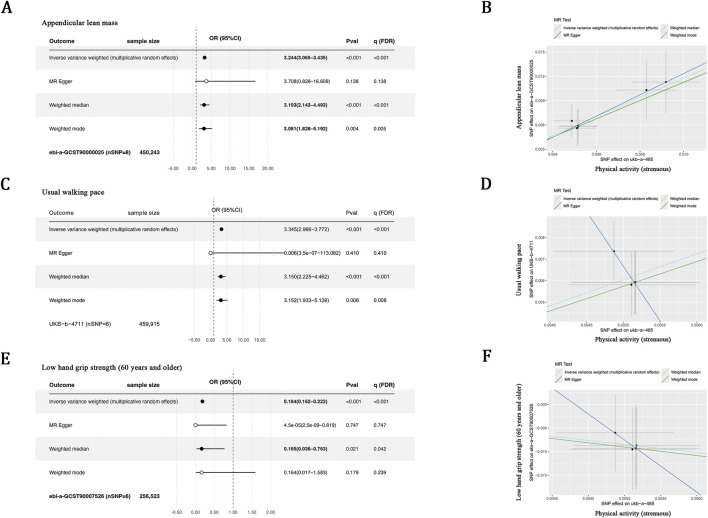
Mendelian randomization analysis of the causal effects of physical activity on sarcopenia-related traits. **(A,C,E)** Forest plots showing causal estimates derived from four MR methods (IVW, MR-Egger, weighted median, and weighted mode), with corresponding odds ratios (ORs) and 95% CI. **(B,D,F)** Scatter plots illustrating the associations between SNP–exposure and SNP–outcome, with fitted regression lines representing causal estimates obtained from each MR method.

Physical exercise is a well-recognized intervention for the prevention of muscle atrophy (Lu et al., 2021). To evaluate the potential causal effects of physical activity on traits associated with sarcopenia, a MR analysis was conducted, focusing on appendicular lean mass, usual walking pace, and low hand grip strength (aged ≥60 years). The analysis revealed a significant positive causal relationship between physical activity and appendicular lean mass (IVW OR = 3.24, 95% CI 3.07–3.44, FDR <0.001; Figures 3A,B) as well as usual walking pace (IVW OR = 3.35, 95% CI 2.97–3.77, FDR <0.001; Figures 3C,D). In contrast, increased physical activity was causally linked to a decreased risk of low hand grip strength (IVW OR = 0.18, 95% CI 0.15–0.22, FDR <0.001; Figures 3E,F). Throughout the analyses, no substantial heterogeneity or horizontal pleiotropy was detected after correction for MR-PRESSO–identified outliers, supporting the robustness of the causal estimates.

Given the observed associations among physical activity, circulating GDF11, and sarcopenia, mediation analysis was conducted. Results indicated that physical activity exerted a significant direct effect on sarcopenia (B = −0.87, *P* = 0.009). Notably, a significant indirect effect mediated by decreased circulating GDF11 levels was also identified (B = −0.13; 95% CI, −0.37 to −0.01). This mediation effect accounted for 13.0% of the total effect, raising the possibility that the downregulation of circulating GDF11 might contribute to the beneficial impact of physical activity on sarcopenia (Table 7).

**TABLE 7:** Mediating effect of GDF11 on the association between physical activity and sarcopenia (PROCESS Model).

X	M	Y	Total effect X→Y	Direct effect	*P*	Indirect effect	Indirect effect proportion
Physical activity	GDF11	Sarcopenia	−1.00	−0.87 (-1.52∼ −0.21)	0.009	−0.13 (−0.37∼ −0.01)	13.0%

### Skeletal muscle is unlikely the primary source of circulating GDF11 in sarcopenia

**FIGURE 4:**
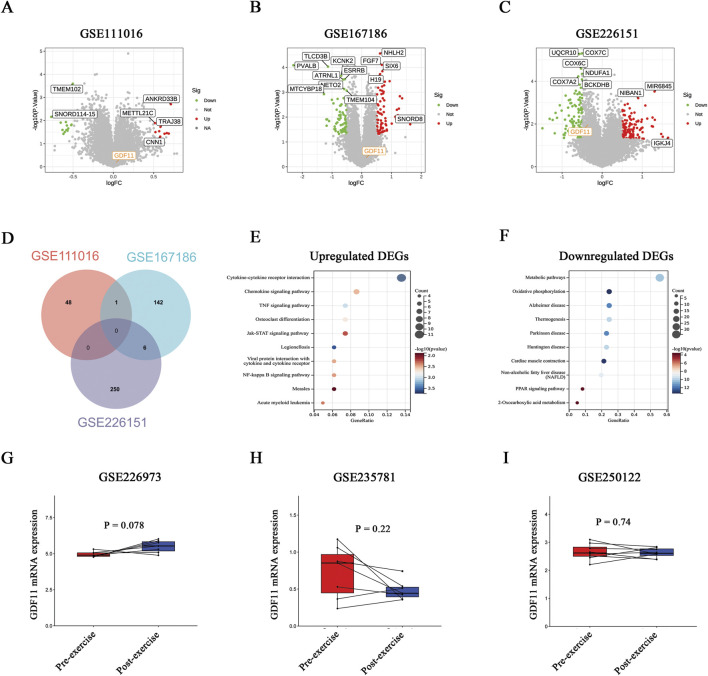
Transcriptomic analysis of GDF11 expression in sarcopenia and exercise-related muscle samples. **(A–C)** Volcano plots of DEGs between sarcopenic and non-sarcopenic groups in datasets GSE111016, GSE167186, and GSE226151. Red and green points indicate upregulated and downregulated genes, respectively. **(D)** Venn diagram depicting the overlap of DEGs among the three datasets. **(E,F)** KEGG pathway enrichment analysis of the pooled DEGs, showing pathways associated with upregulated **(E)** and downregulated **(F)** genes. **(G–I)** GDF11 mRNA levels in skeletal muscle before and after exercise across datasets GSE226973, GSE235781, and GSE250122. Statistical significance was evaluated using paired t-tests.

To assess GDF11 expression in the context of sarcopenia, three independent transcriptomic datasets (GSE111016, GSE167186, and GSE226151) were analyzed. No significant differences in GDF11 mRNA expression were observed between sarcopenic and non-sarcopenic individuals across all datasets (Figures 4A–C). Intersection analysis of differentially expressed genes (DEGs) revealed minimal overlap among the three datasets, indicating substantial molecular heterogeneity associated with sarcopenia (Figure 4D). Given this heterogeneity, DEGs were subsequently integrated across datasets, yielding a total of 452 genes, including 238 upregulated and 214 downregulated genes. Functional enrichment analysis further showed that upregulated genes were predominantly enriched in inflammatory pathways, such as cytokine–cytokine receptor interaction, TNF signaling, and JAK–STAT signaling, whereas downregulated genes were mainly associated with metabolic pathways and oxidative phosphorylation (Figures 4E,F).

To further examine whether exercise influences GDF11 expression in skeletal muscle, three exercise-intervention transcriptomic datasets (GSE226973, GSE235781, and GSE250122) were analyzed. Consistently, no significant changes in GDF11 mRNA levels were detected before and after exercise interventions (Figures 4G–I). Collectively, these findings suggest that the elevated circulating GDF11 observed in sarcopenia is unlikely to originate primarily from skeletal muscle, and that muscular GDF11 expression appears largely unresponsive to exercise.

### Exogenous GDF11 induces muscle atrophy signaling in C2C12 myotubes

**FIGURE 5:**
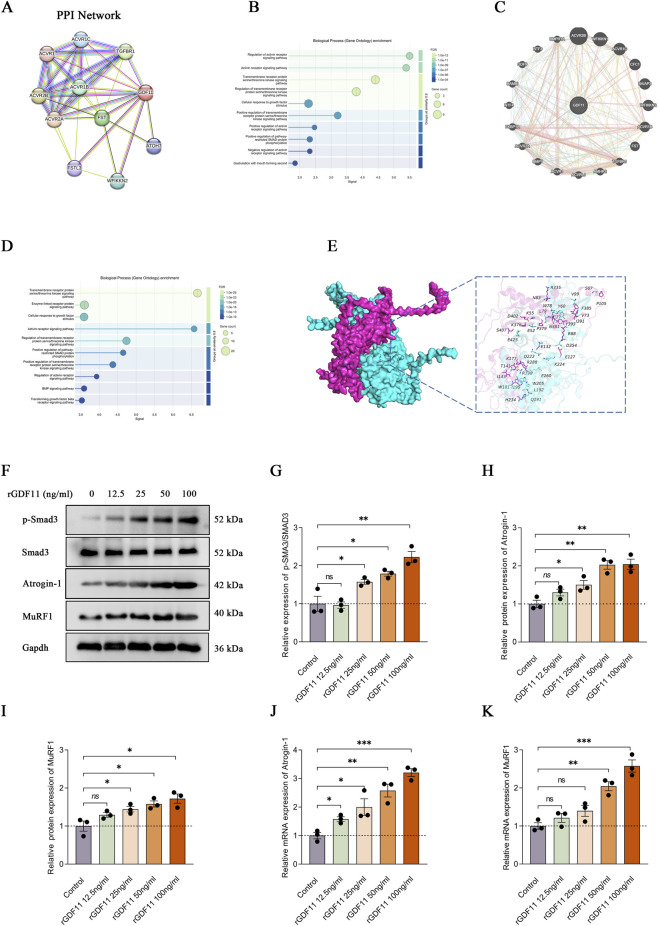
Exogenous GDF11 induces muscle atrophy signaling in C2C12 myotubes. **(A)** GDF11 PPI network constructed using STRING. **(B)** GO-BP enrichment analysis derived from the STRING dataset. **(C)** GDF11 PPI network constructed using GeneMANIA. **(D)** GO-BP enrichment analysis derived from the GeneMANIA dataset. **(E)** Protein–protein docking simulation of the GDF11–ACVR2B complex. **(F)** Representative Western blot images of p-SMAD3, SMAD3, Atrogin-1, MuRF1, and Gapdh in C2C12 myotubes treated with rGDF11 (0–100 ng/mL) for 48 h. **(G)** Densitometric quantification of the p-SMAD3/SMAD3 protein ratio. **(H)** Densitometric quantification of Atrogin-1 protein expression. **(I)** Densitometric quantification of MuRF1 protein expression. **(J,K)** Relative mRNA expression of Atrogin-1 and MuRF1. Data are mean ± SEM. *ns*, not significant, *P < 0.05, **P < 0.01, ***P < 0.001 versus control; *n* = 3.

Given the elevated circulating GDF11 levels observed in patients with sarcopenia, we sought to explore the mechanistic basis underlying its potential pro-atrophic effects. PPI network analysis identified a prominent interaction cluster centered on GDF11, featuring strong associations with activin receptors (ACVRs), particularly activin A receptor type 2B (ACVR2B), a well-established regulator of skeletal muscle mass and muscle atrophy (Hulmi et al., 2018; Lee et al., 2020) (Figures 5A,C). Consistent with this, GO-BP enrichment analysis revealed that GDF11-associated proteins were significantly enriched in ACVRs, the TGF-β pathway, and SMAD phosphorylation cascades (Figures 5B,D). To further substantiate these findings at the structural level, protein–protein docking simulations demonstrated a high-affinity interaction between GDF11 and ACVR2B (binding free energy: −275.86 kcal/mol), stabilized by multiple hydrogen bonds, salt bridges, and hydrophobic interactions at the binding interface (Figure 5E).

Given that ACVR2B is a well-established upstream receptor that predominantly transduces signals via SMAD2/3 phosphorylation (Abazarikia et al., 2025), and that SMAD3 activation is a well-established driver of muscle atrophy through transcriptional induction of the E3 ubiquitin ligases MuRF1 and Atrogin-1 (Frohlich et al., 2022; Peris-Moreno et al., 2021; Roh et al., 2019; Lan et al., 2024), we next sought to experimentally validate the functional impact of GDF11 on myotube catabolic signaling. Differentiated myotubes were exposed to recombinant GDF11 (rGDF11; 0–100 ng/mL) for 48 h. Immunoblotting revealed a robust and dose-dependent increase in SMAD3 phosphorylation, without appreciable changes in total SMAD3 levels (Figures 5F,G). Consistently, rGDF11 treatment led to a progressive upregulation of the muscle-specific ubiquitin ligases MuRF1 and Atrogin-1 at both the protein and mRNA levels (Figures 5H–K). Together, these findings demonstrate that exogenous GDF11 activates the ACVR2B–SMAD3 signaling cascade and promotes a transcriptional program characteristic of muscle atrophy, implicating enhanced ubiquitin–proteasome–mediated proteolysis as a downstream effector mechanism.

## Discussion

Circulating proteins are increasingly recognized as important biomarkers and therapeutic targets for age-related pathologies (Argentieri et al., 2024). Here, we provide evidence that circulating GDF11 levels increase with aging and are independently associated with sarcopenia, with systemic concentrations inversely correlated with physical activity, suggesting potential modulation by lifestyle factors. In contrast, transcriptomic datasets demonstrated that GDF11 mRNA expression in skeletal muscle remains largely unchanged regardless of sarcopenia or exercise status, indicating that circulating GDF11 is likely regulated independently of local muscle transcription. Despite this apparent dissociation, exposure to exogenous GDF11 induced the expression of proteolysis-related genes in myotubes, supporting a functional role for circulating GDF11 in activating muscle catabolic signaling and perturbing muscle homeostasis.

GDF11, a member of the TGF-β superfamily, also known as bone morphogenetic protein 11 (BMP11), plays an essential role in mammalian development (Habibi et al., 2024). Early studies proposed that circulating GDF11 levels decline with age and that exogenous supplementation could reverse age-associated dysfunction in the heart, skeletal muscle, and brain (Loffredo et al., 2013; Sinha et al., 2014; Katsimpardi et al., 2014). Subsequent studies based on animal and cell-based models have challenged this view, demonstrating that circulating GDF11 levels increase with age (Egerman et al., 2015; Poggioli et al., 2016; Liu et al., 2018) and fail to rejuvenate aged skeletal muscle satellite cells (Hinken et al., 2016) or reverse pathological cardiac hypertrophy (Smith et al., 2015). In this study, circulating GDF11 levels increased with aging and were independently associated with sarcopenia. Mechanistically, our data indicate that GDF11 engages activin type II receptors—particularly ACVR2B, a well-established regulator of skeletal muscle mass and atrophy—to activate downstream SMAD2/3 signaling (Hulmi et al., 2018; Lee et al., 2020; Abazarikia et al., 2025). Activation of this pathway promotes the transcription of the E3 ubiquitin ligases MuRF1 and Atrogin-1, central mediators of ubiquitin–proteasome–dependent muscle proteolysis and atrophy (Frohlich et al., 2022; Peris-Moreno et al., 2021; Roh et al., 2019; Lan et al., 2024). The physiological relevance of GDF11 is supported by prior *in vivo* studies demonstrating that sustained elevation of GDF11, through genetic overexpression or exogenous exposure, activates SMAD-dependent catabolic programs and suppresses muscle regeneration, reduces bone mass, and accelerates functional decline in cardiac and skeletal muscle, whereas functional inhibition of GDF11 via its propeptide promotes skeletal muscle hypertrophy (Jin et al., 2019; Egerman et al., 2015; Wang C. et al., 2024; Hsia et al., 2022; Hammers et al., 2017; Liu et al., 2016; Zimmers et al., 2017; Fife et al., 2018). Beyond this core SMAD-dependent mechanism, crosstalk between SMAD signaling and the FOXO3a and NF-κB pathways—both well-established regulators of muscle atrophy—may further potentiate the overall catabolic response (Luo, 2017). Taken together, despite reported beneficial effects in other tissues (Moigneu et al., 2023; Wu et al., 2024; Yin et al., 2021), the available evidence supports a predominant role for GDF11 as a catabolic regulator in skeletal muscle.

We also acknowledge the potential contribution of growth and differentiation factor 8 (GDF8, myostatin), given its high structural homology with GDF11. The analytical approach used in this study is consistent with that of previous investigations (Moigneu et al., 2023; Xing et al., 2024; Cohen et al., 2025); however, accurately distinguishing these closely related ligands using conventional immunoassays remains a well-recognized technical challenge in the field (Rodgers and Eldridge, 2015; Harper et al., 2016; Ochsner et al., 2019). To definitively distinguish GDF11 from GDF8, future studies should incorporate liquid chromatography–tandem mass spectrometry (LC–MS/MS), which enables the identification of unique peptide signatures arising from subtle structural differences between the two proteins (Schafer et al., 2016). In addition, quantification of their respective propeptides represents a complementary strategy, as these regions exhibit substantially lower sequence homology than the mature ligands (Olson et al., 2015). Adoption of such rigorous analytical approaches will be essential for delineating the distinct pathophysiological roles of GDF11 and GDF8 in muscle aging.

GDF11 is ubiquitously expressed during embryogenesis across multiple tissues, including the central nervous system, skeletal muscle, heart, kidneys, and bone (Zhang et al., 2017). In adult organisms, its expression declines substantially but remains detectable in skeletal and cardiac muscle, liver, adipose tissue, and the nervous system (Machelak et al., 2023; Uhlén et al., 2015). Despite this broad tissue distribution, the precise anatomical sources of circulating GDF11 remain incompletely defined. Platelets have been proposed as a potential reservoir owing to their high GDF11 content (Bueno et al., 2016), and several organs—including the heart, lung, and kidney—have been shown to possess secretory capacity (Leitner et al., 2017; von Haehling et al., 2009). In murine models, modulation of myocardial GDF11 expression resulted in modest alterations in plasma GDF11 levels (approximately 0.8- to 1.3-fold) without evidence of systemic toxicity (Harper et al., 2018). A new finding of our study is the dissociation between local skeletal muscle expression and systemic circulating GDF11 levels. Transcriptomic analyses revealed that GDF11 mRNA expression in skeletal muscle remained largely unchanged regardless of sarcopenia status or physical activity levels. This absence of a transcriptional response suggests that the elevated circulating GDF11 observed in sarcopenia is likely derived predominantly from extra-muscular sources rather than skeletal muscle itself. Given the invasive nature and ethical constraints associated with obtaining visceral organ biopsies in older adults without clinical indications, direct validation of tissue-specific contributions in human cohorts is not currently feasible. Future studies using animal models should integrate tissue-specific genetic perturbation strategies with isotope-based protein tracing to identify the primary tissues contributing to circulating GDF11 under physiological and pathological conditions.

Physical activity is extensively acknowledged as an effective intervention for the prevention or mitigation of muscle atrophy, particularly in cases of inactivity- or disuse-related sarcopenia (Arif et al., 2025). In our study, logistic regression analysis identified increased physical activity as a significant protective factor against sarcopenia, a finding further supported by Mendelian randomization, which indicated a causal benefit. Notably, mediation analysis suggested that circulating GDF11 may partially mediate this protective effect. Consistent with this, an 8-week multimodal training program in older adults with sarcopenia was shown to significantly reduce circulating GDF11 levels while enhancing body composition (Bagheri et al., 2020). In contrast, high-intensity endurance exercise in healthy young adults did not alter systemic GDF11 levels, although a decrease was observed in cerebrospinal fluid (Schön et al., 2023). To investigate local molecular responses, we analyzed skeletal muscle GDF11 mRNA expression across various exercise-intervention datasets and found no significant differences among the different modalities. Interestingly, animal studies have indicated that moderate exercise increases GDF11 mRNA levels in the slow-twitch muscles of aged mice, although no corresponding changes in protein levels were detected (Lee et al., 2019). These discrepancies imply that the regulatory relationship between physical activity and GDF11 may differ between systemic circulation and local muscle transcription. The mediation analysis conducted in this study was exploratory and cross-sectional, limiting the ability to draw definitive causal conclusions. Therefore, future prospective longitudinal studies are necessary to ascertain whether physical activity influences circulating GDF11 levels and to identify the specific exercise modalities (e.g., resistance, endurance, or combined training) that may be responsible for these changes.

This study is subject to several limitations. Firstly, the generalizability of our findings is restricted due to the single-center design and relatively modest sample size. Secondly, the cross-sectional nature of the data limits the mediation analysis related to physical activity to an exploratory level, precluding definitive causal inferences. Thirdly, we recognize the methodological challenge inherent in antibody cross-reactivity between GDF11 and GDF8; future research utilizing mass spectrometry–based proteomic techniques will be necessary for precise differentiation. From a mechanistic standpoint, while we demonstrate that exogenous GDF11 activates canonical catabolic signaling pathways *in vitro*, translating these cellular observations into a therapeutic context will necessitate further *in vivo* validation. Lastly, the specific tissue sources contributing to elevated circulating GDF11 remain unidentified due to practical constraints in clinical tissue sampling.

## Conclusion

In this study, we observe that circulating GDF11 is elevated in sarcopenia and may partially mediate the protective effects of physical activity. This relationship appears to be uncoupled from skeletal muscle GDF11 transcription, pointing to regulatory mechanisms beyond local muscle expression. In combination with our finding that exogenous GDF11 can activate catabolic signaling in muscle cells, these results suggest a potential role for circulating GDF11 in age-related muscle atrophy.

## Data Availability

Publicly available datasets were analyzed in this study, and these data can be accessed from the following sources: GEO database: https://www.ncbi.nlm.nih.gov/geo/, OpenGWAS database: https://gwas.mrcieu.ac.uk/, GeneMANIA database: https://genemania.org/, STRING database: https://string-db.org/.

## References

[B1] Abazarikia A. So W. Luan Y. Kattamuri C. Thompson T. B. Kim S. Y. (2025). Pancreatic damage in ovarian cancer-associated cachexia is driven by activin A signalling. J. Cachexia Sarcopenia Muscle 16 (5), e70096. 10.1002/jcsm.70096 41071262 PMC12512902

[B2] Argentieri M. A. Xiao S. Bennett D. Winchester L. Nevado-Holgado A. J. Ghose U. (2024). Proteomic aging clock predicts mortality and risk of common age-related diseases in diverse populations. Nat. Medicine 30 (9), 2450–2460. 10.1038/s41591-024-03164-7 39117878 PMC11405266

[B3] Arif I. Rasheed A. Nazeer S. Shahid F. (2025). Physiological and morphological impact of physical activity and nutritional interventions to offset disuse-induced skeletal muscle atrophy. Eur. Journal Translational Myology. 2025 Apr 15. Eng. Epub 2025/04/15 35, 13177. 10.4081/ejtm.2025.13177 40231413 PMC12265418

[B4] Bagheri R. Moghadam B. H. Church D. D. Tinsley G. M. Eskandari M. Moghadam B. H. (2020). The effects of concurrent training order on body composition and serum concentrations of follistatin, myostatin and GDF11 in sarcopenic elderly men. Exp. Gerontology 133, 110869. 10.1016/j.exger.2020.110869 32035222

[B5] Bueno J. L. Ynigo M. de Miguel C. Gonzalo-Daganzo R. M. Richart A. Vilches C. (2016). Growth differentiation factor 11 (GDF11) - a promising anti-ageing factor - is highly concentrated in platelets. Vox Sanguinis 111 (4), 434–436. 10.1111/vox.12438 27509407

[B6] Chen L. K. Woo J. Assantachai P. Auyeung T. W. Chou M. Y. Iijima K. (2020). Asian working group for sarcopenia: 2019 consensus update on sarcopenia diagnosis and treatment. J. Am. Med. Dir. Assoc. 21 (3), 300–307. 10.1016/j.jamda.2019.12.012 32033882

[B7] Cohen O. S. Sinha M. Wang Y. Daman T. Li P. C. Deatherage C. (2025). Recombinant GDF11 promotes recovery in a rat permanent ischemia model of subacute stroke. Stroke 56 (4), 996–1009. 10.1161/strokeaha.124.049908 39909827 PMC11932786

[B8] Egerman M. A. Cadena S. M. Gilbert J. A. Meyer A. Nelson H. N. Swalley S. E. (2015). GDF11 increases with age and inhibits skeletal muscle regeneration. Cell Metab. 22 (1), 164–174. 10.1016/j.cmet.2015.05.010 26001423 PMC4497834

[B9] Egerman M. A. Zhang Y. Donne R. Xu J. Gadi A. McEwen C. (2025). ActRII or BMPR ligands inhibit skeletal myoblast differentiation, and BMPs promote heterotopic ossification in skeletal muscles in mice. Skelet. Muscle 15 (1), 4. 10.1186/s13395-025-00373-7 39994804 PMC11853584

[B10] Fernández-Lázaro D. Garrosa E. Seco-Calvo J. Garrosa M. (2022). Potential satellite cell-linked biomarkers in aging skeletal muscle tissue: proteomics and proteogenomics to monitor sarcopenia. Proteomes 10 (3). 10.3390/proteomes10030029 35997441 PMC9396989

[B11] Fife E. Kostka J. Kroc Ł. Guligowska A. Pigłowska M. Sołtysik B. (2018). Relationship of muscle function to circulating myostatin, follistatin and GDF11 in older women and men. BMC Geriatrics 18 (1), 200. 10.1186/s12877-018-0888-y 30165829 PMC6117873

[B12] Frohlich J. Kovacovicova K. Raffaele M. Virglova T. Cizkova E. Kucera J. (2022). GDF11 inhibits adipogenesis and improves mature adipocytes metabolic function *via* WNT/β-catenin and ALK5/SMAD2/3 pathways. Cell Proliferation 55 (10), e13310. 10.1111/cpr.13310 35920128 PMC9528760

[B13] Garbern J. Kristl A. C. Bassaneze V. Vujic A. Schoemaker H. Sereda R. (2019). Analysis of Cre-mediated genetic deletion of Gdf11 in cardiomyocytes of young mice. Am. Journal Physiology Heart Circulatory Physiology 317 (1), H201–h212. 10.1152/ajpheart.00615.2018 31125255 PMC6692736

[B14] Habibi P. Falamarzi K. Ebrahimi N. D. Zarei M. Malekpour M. Azarpira N. (2024). GDF11: an emerging therapeutic target for liver diseases and fibrosis. J. Cellular Molecular Medicine 28 (7), e18140. 10.1111/jcmm.18140 38494851 PMC10945076

[B15] Hammers D. W. Merscham-Banda M. Hsiao J. Y. Engst S. Hartman J. J. Sweeney H. L. (2017). Supraphysiological levels of GDF11 induce striated muscle atrophy. EMBO Molecular Medicine 9 (4), 531–544. 10.15252/emmm.201607231 28270449 PMC5376753

[B16] Harper S. C. Brack A. MacDonnell S. Franti M. Olwin B. B. Bailey B. A. (2016). Is growth differentiation factor 11 a realistic therapeutic for aging-dependent muscle defects? Circulation Research 118 (7), 1143–1150. 10.1161/circresaha.116.307962 27034276 PMC4829942

[B17] Harper S. C. Johnson J. Borghetti G. Zhao H. Wang T. Wallner M. (2018). GDF11 decreases pressure overload-induced hypertrophy, but can cause severe cachexia and premature death. Circulation Research 123 (11), 1220–1231. 10.1161/circresaha.118.312955 30571461 PMC6309347

[B18] Hemani G. Tilling K. Davey Smith G. (2017). Orienting the causal relationship between imprecisely measured traits using GWAS summary data. PLoS Genetics 13 (11), e1007081. 10.1371/journal.pgen.1007081 29149188 PMC5711033

[B19] Hinken A. C. Powers J. M. Luo G. Holt J. A. Billin A. N. Russell A. J. (2016). Lack of evidence for GDF11 as a rejuvenator of aged skeletal muscle satellite cells. Aging Cell 15 (3), 582–584. 10.1111/acel.12475 27139744 PMC4854912

[B20] Hsiao Y. T. Shimizu I. Yoshida Y. Minamino T. (2022). Role of circulating molecules in age-related cardiovascular and metabolic disorders. Inflamm. Regeneration 42 (1), 2. 10.1186/s41232-021-00187-2 35012677 PMC8744343

[B21] Hulmi J. J. Nissinen T. A. Räsänen M. Degerman J. Lautaoja J. H. Hemanthakumar K. A. (2018). Prevention of chemotherapy-induced cachexia by ACVR2B ligand blocking has different effects on heart and skeletal muscle. J. Cachexia Sarcopenia Muscle 9 (2), 417–432. 10.1002/jcsm.12265 29230965 PMC5879968

[B22] Idkowiak-Baldys J. Santhanam U. Buchanan S. M. Pfaff K. L. Rubin L. L. Lyga J. (2019). Growth differentiation factor 11 (GDF11) has pronounced effects on skin biology. PloS One 14 (6), e0218035. 10.1371/journal.pone.0218035 31181098 PMC6557520

[B23] Jin Q. Qiao C. Li J. Xiao B. Li J. Xiao X. (2019). A GDF11/myostatin inhibitor, GDF11 propeptide-Fc, increases skeletal muscle mass and improves muscle strength in dystrophic mdx mice. Skelet. Muscle 9 (1), 16. 10.1186/s13395-019-0197-y 31133057 PMC6537384

[B24] Katsimpardi L. Litterman N. K. Schein P. A. Miller C. M. Loffredo F. S. Wojtkiewicz G. R. (2014). Vascular and neurogenic rejuvenation of the aging mouse brain by young systemic factors. Sci. (New York, NY) 344 (6184), 630–634. 10.1126/science.1251141 24797482 PMC4123747

[B25] Kim D. Y. Kang Y. H. Kang M. K. (2025). Umbelliferone attenuates diabetic sarcopenia by modulating mitochondrial quality and the ubiquitin-proteasome system. Phytomedicine 144, 156930. 10.1016/j.phymed.2025.156930 40483791

[B26] Kraler S. Balbi C. Vdovenko D. Lapikova-Bryhinska T. Camici G. G. Liberale L. (2023). Circulating GDF11 exacerbates myocardial injury in mice and associates with increased infarct size in humans. Cardiovasc. Research 119 (17), 2729–2742. 10.1093/cvr/cvad153 37742057 PMC10757585

[B27] Lan X. Q. Deng C. J. Wang Q. Q. Zhao L. M. Jiao B. W. Xiang Y. (2024). The role of TGF-β signaling in muscle atrophy, sarcopenia and cancer cachexia. General Comparative Endocrinology 353, 114513. 10.1016/j.ygcen.2024.114513 38604437

[B28] Lee M. Oikawa S. Ushida T. Suzuki K. Akimoto T. (2019). Effects of exercise training on growth and differentiation factor 11 expression in aged mice. Front. Physiology 10, 970. 10.3389/fphys.2019.00970 31417428 PMC6684741

[B29] Lee S. J. Lehar A. Meir J. U. Koch C. Morgan A. Warren L. E. (2020). Targeting myostatin/activin A protects against skeletal muscle and bone loss during spaceflight. Proc. Natl. Acad. Sci. U. S. A. 117 (38), 23942–23951. 10.1073/pnas.2014716117 32900939 PMC7519220

[B30] Lehallier B. Gate D. Schaum N. Nanasi T. Lee S. E. Yousef H. (2019). Undulating changes in human plasma proteome profiles across the lifespan. Nat. Medicine 25 (12), 1843–1850. 10.1038/s41591-019-0673-2 31806903 PMC7062043

[B31] Leitner L. M. Wilson R. J. Yan Z. Gödecke A. (2017). Reactive oxygen species/nitric oxide mediated inter-organ communication in skeletal muscle wasting diseases. Antioxidants and Redox Signaling 26 (13), 700–717. 10.1089/ars.2016.6942 27835923 PMC5421600

[B32] Lin S. Zhong L. Chen J. Zhao Z. Wang R. Zhu Y. (2023). GDF11 inhibits adipogenesis of human adipose-derived stromal cells through ALK5/KLF15/β-catenin/PPARγ Cascade. Heliyon 9 (2), e13088. 10.1016/j.heliyon.2023.e13088 36755591 PMC9900277

[B33] Liu W. Zhou L. Zhou C. Zhang S. Jing J. Xie L. (2016). GDF11 decreases bone mass by stimulating osteoclastogenesis and inhibiting osteoblast differentiation. Nat. Communications 7, 12794. 10.1038/ncomms12794 27653144 PMC5036163

[B34] Liu A. Dong W. Peng J. Dirsch O. Dahmen U. Fang H. (2018). Growth differentiation factor 11 worsens hepatocellular injury and liver regeneration after liver ischemia reperfusion injury. FASEB Journal Official Publication Fed. Am. Soc. Exp. Biol. 32 (9), 5186–5198. 10.1096/fj.201800195R 29913561

[B35] Loffredo F. S. Steinhauser M. L. Jay S. M. Gannon J. Pancoast J. R. Yalamanchi P. (2013). Growth differentiation factor 11 is a circulating factor that reverses age-related cardiac hypertrophy. Cell 153 (4), 828–839. 10.1016/j.cell.2013.04.015 23663781 PMC3677132

[B36] Lu L. Mao L. Feng Y. Ainsworth B. E. Liu Y. Chen N. (2021). Effects of different exercise training modes on muscle strength and physical performance in older people with sarcopenia: a systematic review and meta-analysis. BMC Geriatrics 21 (1), 708. 10.1186/s12877-021-02642-8 34911483 PMC8672633

[B37] Luo K. (2017). Signaling cross talk between TGF-β/Smad and other signaling pathways. Cold Spring Harb. Perspectives Biology 9 (1), eng. 10.1101/cshperspect.a022137 27836834 PMC5204325

[B38] Machelak W. Szczepaniak A. Jacenik D. Zielińska M. (2023). The role of GDF11 during inflammation - an overview. Life Sciences 322, 121650. 10.1016/j.lfs.2023.121650 37011872

[B39] Moigneu C. Abdellaoui S. Ramos-Brossier M. Pfaffenseller B. Wollenhaupt-Aguiar B. de Azevedo Cardoso T. (2023). Systemic GDF11 attenuates depression-like phenotype in aged mice *via* stimulation of neuronal autophagy. Nat. Aging 3 (2), 213–228. 10.1038/s43587-022-00352-3 37118117 PMC10154197

[B40] Nederveen J. P. Ibrahim G. Fortino S. A. Snijders T. Kumbhare D. Parise G. (2020). Variability in skeletal muscle fibre characteristics during repeated muscle biopsy sampling in human vastus lateralis. Appl. physiology, Nutr. metabolism = Physiologie appliquee, Nutr. metabolisme 45 (4), 368–375. 10.1139/apnm-2019-0263 32207991

[B41] Ochsner U. A. Green L. S. Rice T. P. Olivas E. Janjic N. Katilius E. (2019). Targeting unique epitopes on highly similar proteins GDF-11 and GDF-8 with modified DNA aptamers. Biochemistry 58 (46), 4632–4640. 10.1021/acs.biochem.9b00760 31638376

[B42] Olson K. A. Beatty A. L. Heidecker B. Regan M. C. Brody E. N. Foreman T. (2015). Association of growth differentiation factor 11/8, putative anti-ageing factor, with cardiovascular outcomes and overall mortality in humans: analysis of the heart and soul and HUNT3 cohorts. Eur. Heart J. 36 (48), 3426–3434. 10.1093/eurheartj/ehv385 26294790 PMC4685178

[B43] Peris-Moreno D. Cussonneau L. Combaret L. Polge C. Taillandier D. (2021). Ubiquitin ligases at the heart of skeletal muscle atrophy control. Mol. Basel, Switz. 26 (2), 407. 10.3390/molecules26020407 33466753 PMC7829870

[B44] Poggioli T. Vujic A. Yang P. Macias-Trevino C. Uygur A. Loffredo F. S. (2016). Circulating growth differentiation factor 11/8 levels decline with age. Circulation Research 118 (1), 29–37. 10.1161/circresaha.115.307521 26489925 PMC4748736

[B45] Prior S. J. Ryan A. S. Blumenthal J. B. Watson J. M. Katzel L. I. Goldberg A. P. (2016). Sarcopenia is associated with lower skeletal muscle capillarization and exercise capacity in older adults. Journals Gerontology Ser. A, Biol. Sciences Medical Sciences 71 (8), 1096–1101. 10.1093/gerona/glw017 26888434 PMC5007615

[B46] Ritchie M. E. Phipson B. Wu D. Hu Y. Law C. W. Shi W. (2015). Limma powers differential expression analyses for RNA-sequencing and microarray studies. Nucleic Acids Research 43 (7), e47. 10.1093/nar/gkv007 25605792 PMC4402510

[B47] Rodgers B. D. Eldridge J. A. (2015). Reduced circulating GDF11 is unlikely responsible for age-dependent changes in mouse heart, muscle, and brain. Endocrinology 156 (11), 3885–3888. 10.1210/en.2015-1628 26372181

[B48] Roh J. D. Hobson R. Chaudhari V. Quintero P. Yeri A. Benson M. (2019). Activin type II receptor signaling in cardiac aging and heart failure. Sci. Translational Medicine 11 (482), eng. 10.1126/scitranslmed.aau8680 30842316 PMC7124007

[B49] Sayer A. A. Cooper R. Arai H. Cawthon P. M. Ntsama Essomba M. J. Fielding R. A. (2024). Sarcopenia. Nat. Reviews Dis. Primers 10 (1), 68. 10.1038/s41572-024-00550-w 39300120

[B50] Schafer M. J. Atkinson E. J. Vanderboom P. M. Kotajarvi B. White T. A. Moore M. M. (2016). Quantification of GDF11 and myostatin in human aging and cardiovascular disease. Cell Metab. 23 (6), 1207–1215. 10.1016/j.cmet.2016.05.023 27304512 PMC4913514

[B51] Schön M. Marček Malenovská K. Nemec M. Alchus Laiferová N. Straka I. Košutzká Z. (2023). Acute endurance exercise modulates growth differentiation factor 11 in cerebrospinal fluid of healthy young adults. Front. Endocrinology 14, 1137048. 10.3389/fendo.2023.1137048 37033257 PMC10073538

[B52] Sharma V. McNeill J. H. (2009). To scale or not to scale: the principles of dose extrapolation. Br. Journal Pharmacology 157 (6), 907–921. 10.1111/j.1476-5381.2009.00267.x 19508398 PMC2737649

[B53] Sinha M. Jang Y. C. Oh J. Khong D. Wu E. Y. Manohar R. (2014). Restoring systemic GDF11 levels reverses age-related dysfunction in mouse skeletal muscle. Science 344 (6184), 649–652. 10.1126/science.1251152 24797481 PMC4104429

[B54] Smith S. C. Zhang X. Zhang X. Gross P. Starosta T. Mohsin S. (2015). GDF11 does not rescue aging-related pathological hypertrophy. Circulation Research 117 (11), 926–932. 10.1161/circresaha.115.307527 26383970 PMC4636963

[B55] Su H. H. Liao J. M. Wang Y. H. Chen K. M. Lin C. W. Lee I. H. (2019). Exogenous GDF11 attenuates non-canonical TGF-β signaling to protect the heart from acute myocardial ischemia-reperfusion injury. Basic Research Cardiology 114 (3), 20. 10.1007/s00395-019-0728-z 30900023

[B56] Sutherland B. A. Hadley G. Alexopoulou Z. Lodge T. A. Neuhaus A. A. Couch Y. (2020). Growth differentiation Factor-11 causes neurotoxicity during ischemia *in vitro* . Front. Neurology 11, 1023. 10.3389/fneur.2020.01023 33013673 PMC7512098

[B57] Uhlén M. Fagerberg L. Hallström B. M. Lindskog C. Oksvold P. Mardinoglu A. (2015). Proteomics. Tissue-based map of the human proteome. Sci. (New York, NY) 347 (6220), 1260419. 10.1126/science.1260419 25613900

[B58] von Haehling S. Lainscak M. Springer J. Anker S. D. (2009). Cardiac cachexia: a systematic overview. Pharmacol. and Therapeutics 121 (3), 227–252. 10.1016/j.pharmthera.2008.09.009 19061914

[B59] Wang C. Liu X. Hu X. Wu T. Duan R. (2024a). Therapeutic targeting of GDF11 in muscle atrophy: insights and strategies. Int. Journal Biological Macromolecules 279 (Pt 3), 135321. 10.1016/j.ijbiomac.2024.135321 39236952

[B60] Wang M. Y. Yang J. M. Wu Y. Li H. Zhong Y. B. Luo Y. (2024b). Curcumin-activated Wnt5a pathway mediates Ca(2+) channel opening to affect myoblast differentiation and skeletal muscle regeneration. J. Cachexia Sarcopenia Muscle 15 (5), 1834–1849. 10.1002/jcsm.13535 38982896 PMC11446719

[B61] Wu T. Hu E. Xu S. Chen M. Guo P. Dai Z. (2021). clusterProfiler 4.0: a universal enrichment tool for interpreting omics data. Innov. Camb. 2 (3), 100141. 10.1016/j.xinn.2021.100141 34557778 PMC8454663

[B62] Wu Q. Fan C. Liu K. Tang J. (2024). GDF11 inhibits the malignant progression of hepatocellular carcinoma *via* regulation of the mTORC1-autophagy axis. Exp. Therapeutic Medicine 27 (6), 252. 10.3892/etm.2024.12540 38682112 PMC11046183

[B63] Xing Y. Ma X. Zhai R. Chen W. Yan H. (2024). GDF11 improves hippocampal neurogenesis and cognitive abilities in diabetic mice by reducing neural inflammation. Brain, Behavior, Immunity 120, 21–31. 10.1016/j.bbi.2024.05.024 38777287

[B64] Yin L. Li N. Jia W. Wang N. Liang M. Yang X. (2021). Skeletal muscle atrophy: from mechanisms to treatments. Pharmacol. Research 172, 105807. 10.1016/j.phrs.2021.105807 34389456

[B65] Zhang Y. Wei Y. Liu D. Liu F. Li X. Pan L. (2017). Role of growth differentiation factor 11 in development, physiology and disease. Oncotarget 8 (46), 81604–81616. 10.18632/oncotarget.20258 29113418 PMC5655313

[B66] Zhao Y. Zhu J. Zhang N. Liu Q. Wang Y. Hu X. (2020). GDF11 enhances therapeutic efficacy of mesenchymal stem cells for myocardial infarction *via* YME1L-mediated OPA1 processing. Stem Cells Translational Medicine 9 (10), 1257–1271. 10.1002/sctm.20-0005 32515551 PMC7519765

[B67] Zimmers T. A. Jiang Y. Wang M. Liang T. W. Rupert J. E. Au E. D. (2017). Exogenous GDF11 induces cardiac and skeletal muscle dysfunction and wasting. Basic Research Cardiology 112 (4), 48. 10.1007/s00395-017-0639-9 28647906 PMC5833306

